# A Novel Locating System for Cereal Plant Stem Emerging Points’ Detection Using a Convolutional Neural Network

**DOI:** 10.3390/s18051611

**Published:** 2018-05-18

**Authors:** Hadi Karimi, Søren Skovsen, Mads Dyrmann, Rasmus Nyholm Jørgensen

**Affiliations:** 1 Department of Biosystems Engineering, Faculty of Agriculture, University of Tabriz, Tabriz 29 Bahman Boulevard, Tabriz 5166616471, Iran; 2 Department of Engineering—Signal Processing, Aarhus University, DK-8000 Aarhus C, Denmark; ssk@eng.au.dk (S.S.); madsdyrmann@eng.au.dk (M.D.); rnj@eng.au.dk (R.N.J.)

**Keywords:** cereal, plants distribution, sowing performance

## Abstract

Determining the individual location of a plant, besides evaluating sowing performance, would make subsequent treatment for each plant across a field possible. In this study, a system for locating cereal plant stem emerging points (PSEPs) has been developed. In total, 5719 images were gathered from several cereal fields. In 212 of these images, the PSEPs of the cereal plants were marked manually and used to train a fully-convolutional neural network. In the training process, a cost function was made, which incorporates predefined penalty regions and PSEPs. The penalty regions were defined based on fault prediction of the trained model without penalty region assignment. By adding penalty regions to the training, the network’s ability to precisely locate emergence points of the cereal plants was enhanced significantly. A coefficient of determination of about 87 percent between the predicted PSEP number of each image and the manually marked one implies the ability of the system to count PSEPs. With regard to the obtained results, it was concluded that the developed model can give a reliable clue about the quality of PSEPs’ distribution and the performance of seed drills in fields.

## 1. Introduction

In precision farming, precise and accurate sowing of crops across a field is desirable. An accurate distribution will minimize competition among plants and provide proper conditions for nutrients and light, leading to a higher yield [1,2,3]. Furthermore, it allows for precise mechanical weeding. Achieving the desired distribution and densities of crops in fields to a large extent depends on the sowing performance of planters [4]. The sowing performance directly relies on each planter’s components’ efficiency, including the seed metering mechanisms, the seed delivery trajectory that leads seeds to the ground and the seed placement components that should place the seeds with the minimum amount of bouncing. Furthermore, the soil type and condition, physical properties of seeds and the speed of operation all affect the performance of planters in accurate seed distribution [5,6]. Besides the performance of the planter, the location of crops after emergence is influenced by the elimination of early crops due to pests or stressful environmental conditions such as too much or a lack of moisture, low temperature, etc. [7]. By determining precise locations of crops, in addition to evaluating sowing performance of planters across a field, map-based automatic weed control and special treatment for each individual plant like spraying would be possible. Moreover, with plant location maps, the environmental factors that affect plants’ distribution can be investigated.

The developed seed evaluation systems, which can only track seeds until the delivery tube of seed drills [8,9], could not provide precise locations of crops in the fields. A way to determine the exact location of crop plants is to distinguish the plant stem emerging point (PSEP), which is defined as a point where a stem of the plant comes out of the soil surface [10]. Several machine vision studies have been conducted in this area: Two-dimensional (2D) machine vision-based algorithms were developed to detect individual plants, count their populations and define plant spacing [4,11,12]. In these methods, mosaicked top-view images of plants were constructed along the crop rows, which were analyzed to determine the individual plant locations. Utilizing 2D machine vision systems has some difficulties in distinguishing individual plants caused by illumination changes and adjacent plants overlapping when seen from the camera viewpoint. Stereo vision systems that provide top-view depth images have the potential of improving the performance of machine vision systems for determining the PSEP. Jin and Tang [13] have demonstrated such an algorithm that utilizes depth information to effectively find the plant skeletons. Thereby, in a field experiment, individual corn plants were identified with 96.7% precision. Centers of plant positions were estimated with accuracies of 74.6% and 62.3%, respectively, for a maximum distance of 1 cm (six pixels) and 5 cm (30 pixels) from the actual centers. Furthermore, in the development of machine vision systems for plant location sensing, capturing side view depth images from crops was taken into consideration [14,15]. The efficiency of the side-view-based system was fortified by the its ability to recognize inclined plants. Nakarmi and Tang [14] presented a light-based 3D system for acquiring side-view depth images. In this system, distance, intensity and amplitude data were obtained in a single shot. Stem center location was calculated by a feature-based image sequencing process. Although, in comparison with regular color-based machine vision systems, the developed light based system did not depend on color information, sensitivity to light condition and restriction to multiple plant sensing remained. The developed system was evaluated on fields with weeds completely removed. The coefficient of determination between manual field measurement and spacing estimation of corn plants was 0.95. Nakarmi and Tang [15] proposed an approach for a side-view depth image acquisition system by analyzing three consecutive images simultaneously. Mistakes were removed by choosing the best-computed distance between the plants at one-time multiple consecutive image acquisition. The multi-view method was useful in removing the effects of reflected light and sensing double plants. Weeds of the test field were eliminated by herbicide a week before image data acquisition. The developed system had estimated the intra-row maize plant distance with a mean and standard deviation error of 1.60 cm and 2.19 cm, respectively.

Plant locating machine vision systems, as was mentioned above, are regularly confronted with several limitations and difficulties, such as color variations caused by shadow formations and glare, daylight changes, trouble segmenting background weeds, coverage of adjacent plant canopies, etc., such that an algorithm developed based on those images would need to perform under the same conditions, like plant size, color and shape, background composition, illumination, etc . In addition, defined systems had been developed to locate crops like corn/maize, which were planted by precision planters. In this kind of sowing system, single seeds are accurately planted in a distinctive inter-row plant spacing. This condition makes image processing techniques without adjacent plants’ interaction much easier; plus, in some cases, the elimination of weeds had been performed before image capturing. In contrast, cereal seeds are planted by seed drills. The random pattern of planting and typically narrow row spacing of seed drills result in many interactions among plants [5]. This may be a big issue for locating cereal plants using regular image processing techniques. However, some attempts had been made for estimating cereal population and density [16,17]. These authors have not found any report about generalized systems for individual cereal plant locating. Seed drill manufacturers and farmers are looking for a generalized sensing system that can acceptably recognize plant location and give them a clue about sowing performance. Pixels in the emergence region of each crop seem to have similar characteristics considering changes in the pixels of the ground and plants. In this study, using a deep learning method based on a convolutional neural network is proposed to create a map of all regions of interest in the images. The main goal is to develop a CNN-based generalized model to detect PSEPs of cereal plants.

## 2. Materials and Methods

### 2.1. Field Experiment

**Figure 1:**
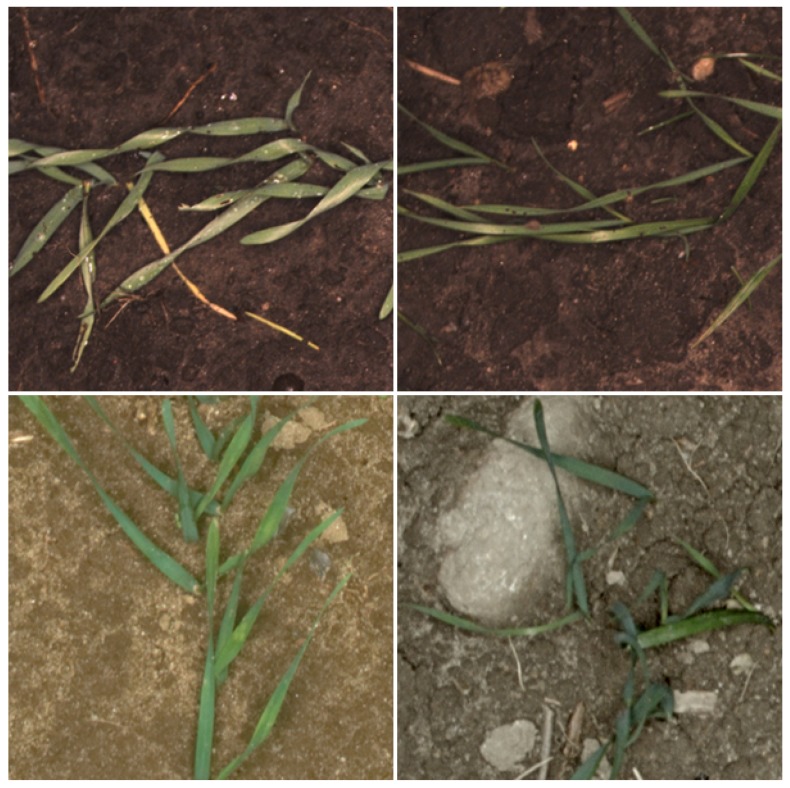
Typical images of Fields 1, 2 and 3 (from left to right, Field 3 and Field 2; bottom, two images from the dataset of Field 1).

Experiments were conducted in several fields in Denmark. Seed drills had been used for sowing cereals seeds like barley and wheat. Images were taken when the cereals were at the early growth stages, but at a time when most of the plants were supposed to have emerged and could be individually distinguishable. In total, 5719 images were acquired. This dataset was divided into three groups, which were Field 1 with 1829 images, Field 2 with 2720 images and Field 3 with 1170 images. Field 1 does not, however, refer to only one field, but is composed of images from several fields. The Field 1 dataset was gathered with the same data acquisition platform from previous projects. Images from this dataset were found to be beneficial for improving the generality of the proposed solution (Figure 1).

### 2.2. Data Acquisition Platform

**Figure 2:**
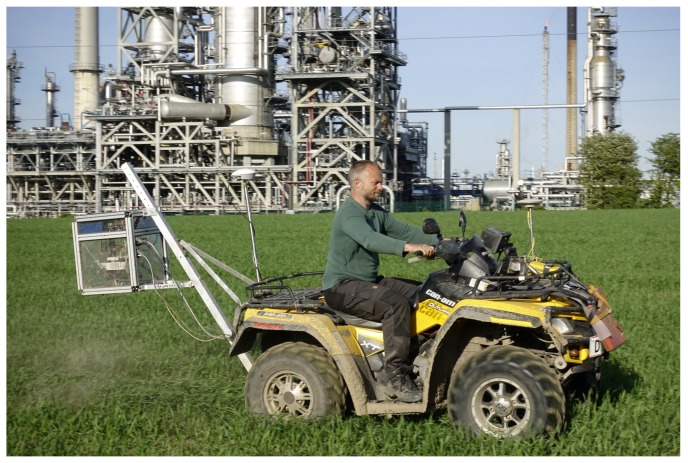
ATV data acquisition platform.

Cereal crop images were captured from an all-terrain vehicle (ATV) (Figure 2), which had been developed by the Signal Processing Group of Aarhus University [18]. The camera setup consisted of a 5.0-Mpixel USB 3.0 camera (Point Grey, GS3-U3-51S5C-C) equipped with a 25-mm lens (Edmund optics, 86-571, Barrington, IL, USA) and a ring flash (AlienBees, ABR800) for lighting support. The ATV data acquisition platform was based on an embedded Linux-based computer (Nvidia TX1) that could capture the images alongside the positions through the mounted RTK-GNSS receiver (Trimble BD920 RTK-GNSS) with a Helix antenna. Image sampling was set to trigger every 10 m based on the Euclidean distance to the last captured image. During the experiment, the ATV started with the perimeter of the fields, then covered the rest of them in a systematic manner between the tramlines, seeking to take a 10 × 10 m${}^{2}$ sampling grid. The resolution of the gathered images was 2456 × 2054 pixels. The camera height was approximately 1.8 m, which provided an image area of 1.11 m × 0.81 m. The ATV wheels were spaced apart in such a way that the effect of the wheels on crops at the rear side would not be captured in the images.

### 2.3. Convolutional Neural Network for Emergence Point Detection

The aim is to develop a model, which can detect the emergence point of each crop-plant in field images, i.e., for each pixel in an input image, the network should associate a label describing whether the pixel is an emergence point or not.

#### 2.3.1. Network Architecture

The convolutional neural network architecture is based on the fully-convolutional neural network with a stride of 8, FCN-8s, by Long et al. [19]. Following the procedure of [19], the 16 layer convolutional neural network, VGG16 [20], is made fully convolutional by transforming the fully-connected layers into convolutional operations, while preserving the learned parameters. The final classification layer is replaced by a 1 × 1 convolutional layer with two kernels, to provide the final distinction between PSEP and the background. The procedure of Long et al. [19] of using pretrained weights of VGG16 [20] from ImageNet was repeated and later retrained for the two-class semantic segmentation problem.

#### 2.3.2. Marking Possible PSEPs

**Figure 3:**
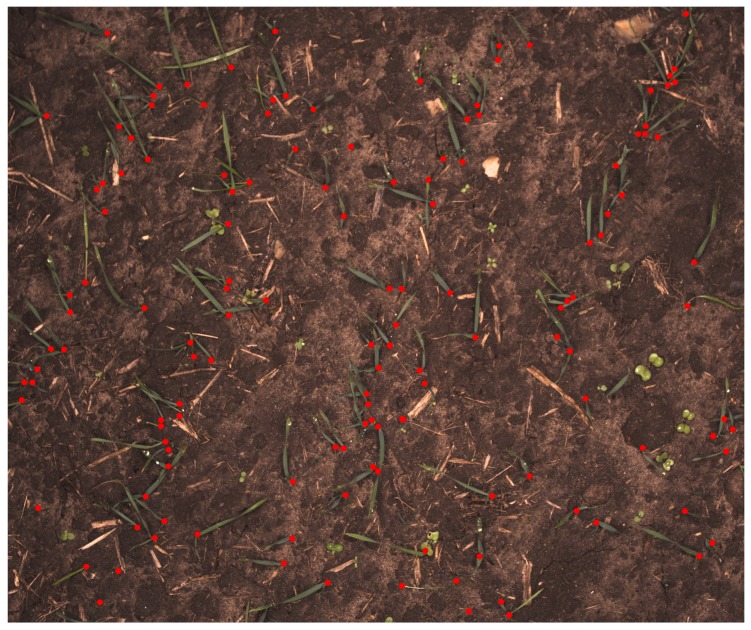
Typical image of manually-marked emergence points of cereals.

The developed network should be able to assign a label to every pixel in an image such that the PSEPs and background can be recognized. To prepare images for the training process, a selected collection of images should be marked manually with recognized PSEPs. The selected images should represent the diversity of PSEPs and the background conditions in fields as much as possible. The greatest effort was made by trained eyes in order to have images of a varied density of plants and backgrounds, including different biomass residues and soils. In this case, 212 images from different fields were chosen and annotated to feed and train a fully-convolutional neural network. An example of an annotated image can be seen in Figure 3. The regions of interest have been labeled by red marks in a separate image layer using GIMP (GNU Image Manipulation Program). The marks are made as complete red circles with a diameter of 20 pixels.

#### 2.3.3. Training

The initial investigation in developing a proper local pixel-based convolutional neural network was done by using the pre-trained FCN-8s with a standard cross-entropy loss function. This network has been implemented in Python using the Tensorflow library. The raw images have been fed to the FCN network as the input and maps of PSEPs as the output. The predictions were compared to the manually-marked images in order to calculate the network’s loss, backpropagate the prediction error and update the parameters. During training, all images were randomly cropped to 1500 × 1500 pixels to reduce overfitting, while being able to fit an image into the memory of an Nvidia TitanX GPU. However, as the network is fully-convolutional, all image sizes bigger than 32 × 32 were accepted. During training, the output of the network was also an image with a size of 1500 × 1500 pixels and two channels reflecting the two classes: PSEPs and background. The network was trained for 40 epochs (an epoch is a single pass of the entire input data) using an Adagrad optimizer with a learning rate of 1×10${}^{-3}$. After 32 and 36 epochs, the learning rate was reduced by a factor of 2.

Although initial training showed promising results, some errors were also observed in the detected PSEPs in the images. Investigating the output images and output softmax (a function in the output layer that converts vectors into class probabilities) revealed that the trained network also responded to tips of leaves, weeds and plant residue as being emerging points. To overcome these faults and improve the network performance, a penalty functionality for the fault prediction region was defined, such that the penalty in training would be increased when misclassifying the predefined penalty regions. The penalty was introduced into the convolutional neural network by extending the loss function, while keeping the network architecture unchanged. The updated cross-entropy loss function with penalty weights included is shown in Equation (1), where *N* is the number of pixels in the input image, *K* is the number of classes, $p_{n}$ is the penalty for the individual pixel, $t_{nk}$ is the one-hot coded target value and $y_{nk}$ is the prediction of the CNN.

(1) $$E=-\frac{1}{K}\sum_{n=1}^{N}\sum_{k=1}^{K}p_{n}t_{nk}ln\left(y_{nk}\right)$$

**Figure 4:**
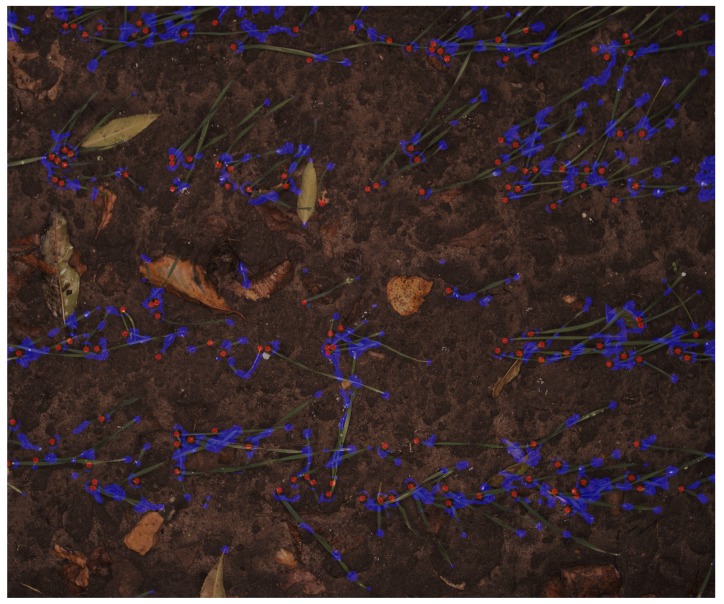
Typical image of penalty regions (blue) along side marked plant stem emerging points (PSEPs) (red).

Penalty regions were created, based on fault prediction regions of the output of the primary trained network. The penalty regions in each image were obtained by subtracting manually-marked images as actual PSEPs from the output of the network. In the output mask, a value of one was assigned to PSEPs, and two was assigned to penalty regions. As the exact pixel positions of PSEPs are hard to define, the markings might be off by a few pixels when comparing them to the computer’s predictions. Therefore, in order to help the computer detect the correct locations, a non-punishing area around the manually-marked points was made. By having this non-punishing area, the computer is guided towards the correct location, as predictions close to the correct locations contribute less to the loss than predictions far from annotated PSEPs. As can be seen from Figure 4, penalty regions were in the tips of the leaves and areas that had some appearance in common with actual PSEPs. The network was then retrained with these new penalty regions in the loss definitions. Penalty weights were applied at three rates of 0, 50 and 75 in the training process. Then, the trained model was evaluated with nine softmax-threshold steps of 0.1, 0.2, 0.3, 0.4, 0.5, 0.6, 0.7, 0.8 and 0.9 percent. Therefore, a collection of 27 models was evaluated (Table 1). Softmax-threshold steps determine how probable it is that the image falls into each target class including the background and PESPs.

#### 2.3.4. Evaluation Method

Due to the heavy class imbalance between PSEP pixels and background pixels, predicting all pixels as background led to a pixel-wise accuracy of approximately 99%, although zero PSEPs were detected. Moving from pixel-wise evaluation metrics towards object detection metrics provides better metrics for evaluating the performance. Recall that the independence of the true prediction of the background would make the results more sensible. The precision calculated the number of true points across all detections. On the other hand, recall estimated the fraction of correct detections relative to all annotations. The recall was sensitive to missed PSEPs, while the precision was sensitive to false detections [21,22,23]. Precision and recall are defined as follows:

(2) $$P=\frac{\mathrm{TP}}{\mathrm{TP}+\mathrm{FP}}$$

(3) $$R=\frac{\mathrm{TP}}{\mathrm{TP}+\mathrm{FN}}$$

where *P* and *R* are the precision and recall, respectively, TP is true point prediction, FP is false prediction and FN is false negative or false prediction background. Furthermore, FN refers to missing points. In regard to the ground-truth annotation, a perfect model would identify all possible PSEPs without any missing points and faults that result in this case in the precision and recall being equal to one. These criteria investigate the performance of a system from two dimensions. High recall could represent a system with the likelihood of false points, and a high precision could have the probability of missing true points. To have a comprehensive judgment about the system with a single quantity, the harmonic mean of the precision and recall was defined with $F_{1}$.

(4) $$F=\frac{2P\times R}{P+R}$$

**Figure 5:**
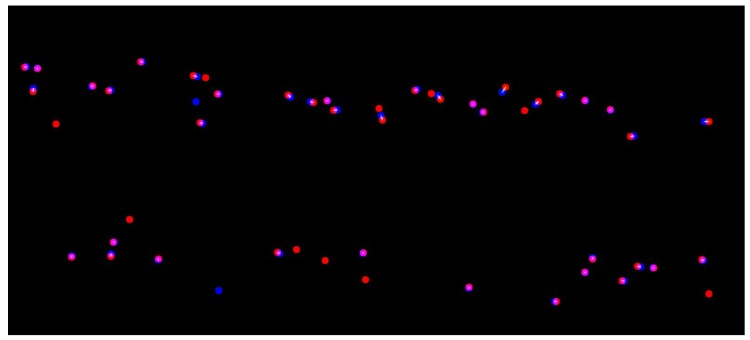
A typical comparison of actual PSEPs and the predicted one in one image. Blue points are false detections; red points are missed detections; and pink points are true detections.

The above-mentioned criteria were estimated by matching the manually-marked images with the output of the developed network. Figure 5 illustrates a typical comparison of marked PSEPs and predicted ones for one image. Overlapped points with pink color and a white line in between were considered as the correct predictions of PSEPs. These points had been counted as TP in the previously-explained formula. Blue points are fault detections of the model, and red ones are marked PSEPs that were missed in the prediction.

## 3. Results

**Figure 6:**
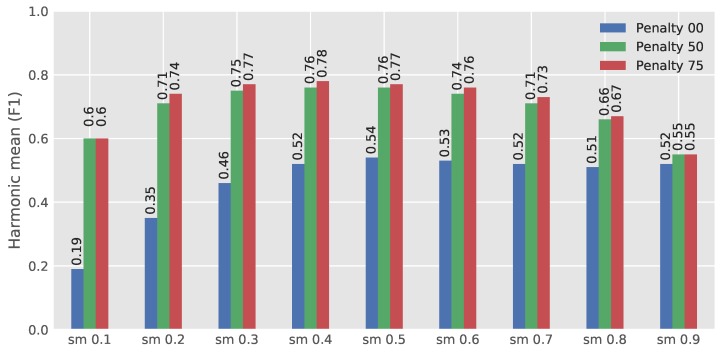
Three steps of the penalty rate in nine steps of the softmax (sm) outputs.

**Figure 7:**
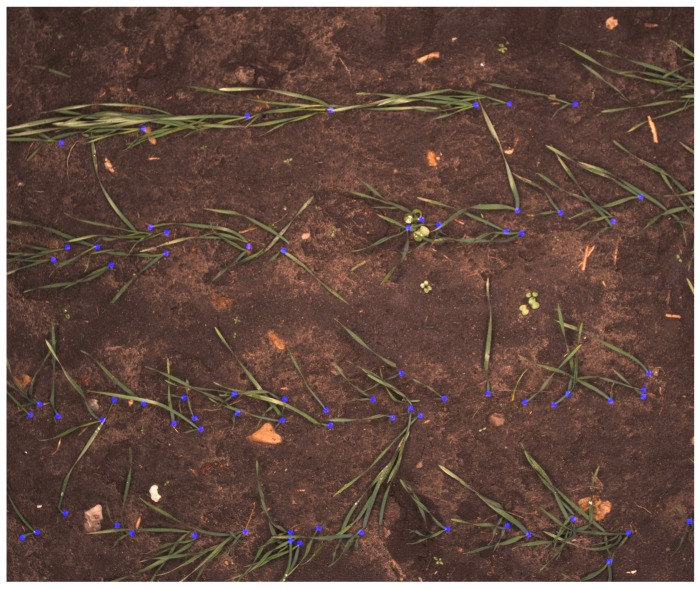
A typical output of the developed network for Field 2.

The network has been trained with three different penalty weights. For each penalty weight, the network was evaluated with nine softmax-thresholds. The best penalty weight and softmax-threshold were found to be 75 and 0.4, respectively, as shown in Figure 6, which illustrates the average of 848 images (212 annotated images with 90 deg rotation and transposing). Figure 6 shows that by adding penalty regions to the training images, the performance of the network is significantly increased. However, exact values were less important, as lowering the softmax down to 0.2 only decreased the $F_{1}$-score slightly. Likewise, when the penalty weight above 50, the $F_{1}$-score stabilized. The network had also been trained with penalty weights of 100 and 125, but the network did not converge to useful results with these settings. This was likely because the penalty regions were located near the actual emergence points, whereby the network was being penalized too much when approaching the actual emergence points. A typical output of the developed network for an untrained image from Field 2 is shown in Figure 7.

### 3.1. Selecting Images from the Dataset for Evaluation

**Figure 8:**
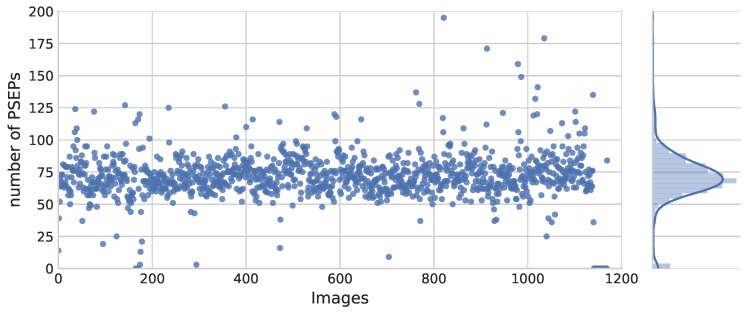
Distribution of predicted PSEPs in Field 3 (the curve on the right illustrates the quality of the number distribution of PSEPs).

Fields were all planted by seed drills with the particular adjustments in seeds’ distribution. Therefore, images across each field were expected to have approximately the same range of PSEPs. Evaluating the model with a regular randomized selection from the dataset would increase the chance of selecting images with a relatively similar number of PSEPs. However, the goal was to cover all population rates and thereby evaluate the ability of the model to correctly detect PSEPs under various distributions. Figure 8 shows the distribution of predicted PSEPs in Field 3. As can be noticed, most images are neighbors of the same bound of PSEPs.

**Figure 9:**
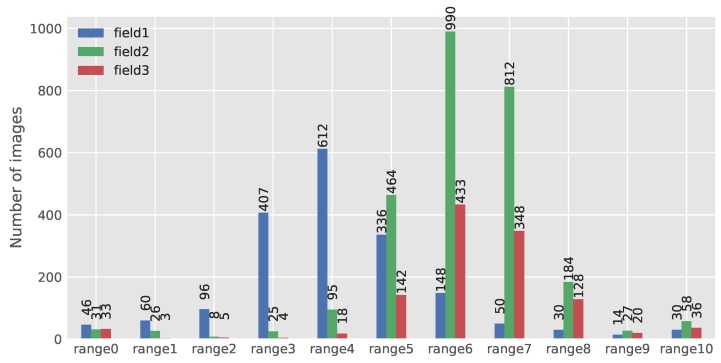
Classifying results of the dataset in Field 1, Field 2 and Field 3. Ranges are Range 0 (PSEPs = 0), Range 1 (0 < PSEPs ≤ 12), Range 2 (12 < PSEPs ≤ 24), Range 3 (24 < PSEPs ≤ 36), Range 4 (36 < PSEPs ≤ 48), Range 5 (48 < PSEPs ≤ 60), Range 6 (60 < PSEPs ≤ 72), Range 7 (72 < PSEPs ≤ 84), Range 8 (84 < PSEPs ≤ 96), Range 9 (96 < PSEPs ≤ 108), Range 10 (108 < PSEPs).

To reach this goal, images from all datasets were fed to the developed network, and the corresponding number of PSEPs for each image were estimated. To have a randomly-chosen image in regard to the number of predicted points in all variations, data were classified into eleven ranges. In this case, the average number of PSEPs in all fields was calculated, and afterward, five steps from the average value were taken up and down. The average number of PSEPs was about 60, which resulted in ranges of 0–10 with a bandwidth of 12. The classifying results of the dataset in Field 1, Field 2 and Field 3 are illustrated in Figure 9. Most of the images in Fields 2 and 3 were classified in the range of 6–7. By knowing the predefined adjustment of seed drills before planting (kilograms per hectare), the performance of the seed drill in each field could be judged. The numbers of PSEPs with further pixel calibration could be considered as the density of crops per unit area.

### 3.2. Evaluating the Developed Network

**Figure 10:**
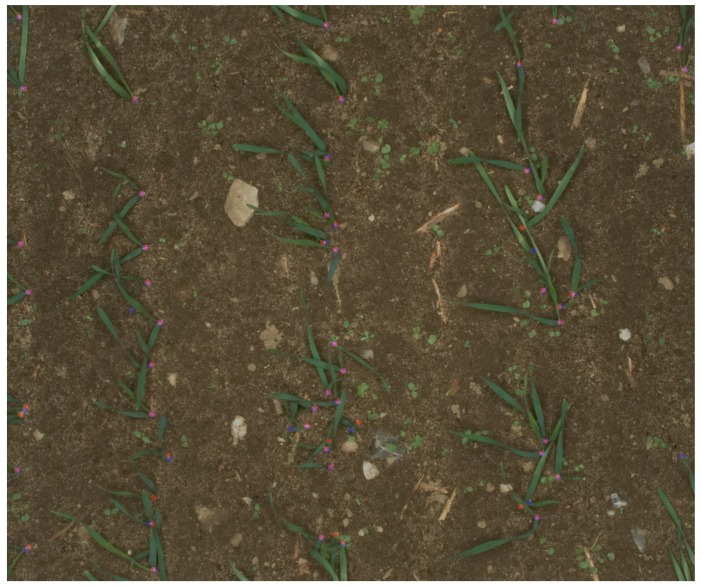
Typical result for images in Field 1 and in Range 5. Blue points are false detections; red points are missed detections; and pink points are true detections.

From each field, in each range, an image was randomly selected for evaluating the model with the ground truth annotation. Therefore, PSEPs for 33 images were marked. These images were fed to the network, and its outputs were compared with the manually-marked ground truth. Typical results for images in Field 1 and in Range 5 are shown in Figure 10. In Figure 10, blue points refer to false predictions by the model (six can be found here); red points refer to missed points (seven can be found here); and pink points are the true prediction of the network (48 points can be found here).

**Figure 11:**
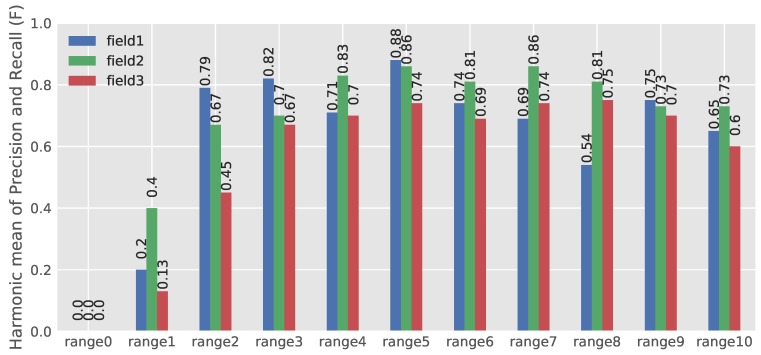
Estimation results of the harmonic mean of precision and recall (F).

The estimation result of the harmonic mean of precision and recall (F) is illustrated in Figure 11. About 74 percent of Field 1 images were classified in Ranges 3, 4 and 5 (Figure 9). The performance of the network in these ranges with 82, 71 and 88 percent for F is quite acceptable. Eight three percent of the images of Field 2 were placed in Ranges 5, 6 and 7. The corresponding *F* was estimated to be 86, 81 and 86, respectively, which represents the good performance of the model in this field. In Field 3, most images, about 79 percent, were classified in Ranges 5, 6 and 7. The calculated *F* for these ranges was 74, 69 and 74 percent, which are interpreted to be relatively acceptable results. The model performance in Field 2 was better than Field 1 and Field 3. This was caused by the variation of different fields in the dataset of Field 1 and the formation of crops in Field 3. Crops in Field 3 had a wider width and more canopy coverage, which made finding possible PSEPs harder even by the trained eye. Therefore, in the process of training, there were more challenges with Field 1 and Field 3.

**Figure 12:**
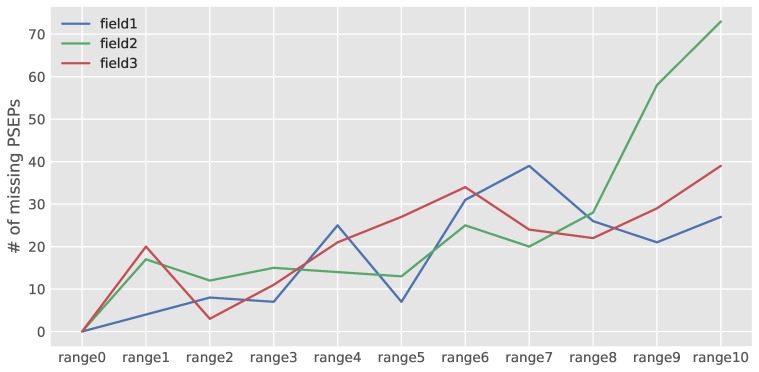
Missing PSEPs of the evaluated images.

Missing PSEPs of the evaluated images that were previously defined as false negatives (FN) in recall Formula (3) are plotted in Figure 12. Figure 12 reveals that by increasing the predicted numbers of PSEPs in each image, the number of missing PSEPs will increase mutually in all fields. More possible PSEPs mean more plants and relevantly more plant density in each image. An increment in plant density would cause more canopy coverage between neighboring plants, making the job of model difficult with respect to identifying probable PSEPs.

**Figure 13:**
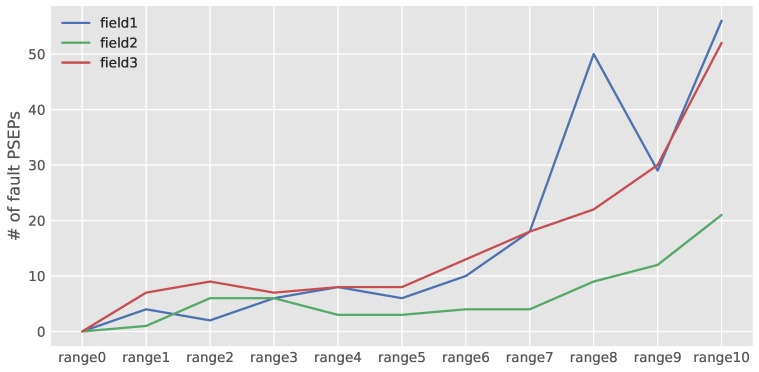
Number of fault predictions in different ranges.

The number of fault prediction in different ranges is plotted Figure 13. The increment in the number of predicted PSEPs caused more faults to occur. By increasing the PSEPs and the number of plants per image, probable regions in the image that would have a character resembling PSEPs will increase. Furthermore, more plants in an image increase the chance of plants and background to be mistakenly recognized as actual PSEPs.

## 4. Discussion

The developed algorithm based on regular machine vision systems would perform appropriately only under predefined conditions like plant size, color, shape, background composition, illumination, etc. Therefore, developing a generalizing machine vision PSEP-locating system based on regular image processing would be troublesome. This would be much more challenging due to the type of cereal cultivation, which was planted in a random pattern and narrow row spacing. In this study, a generalized cereal PSEP-locating system was developed, using deep learning and convolutional neural network techniques. The current approach in comparison with machine vision systems has the important advantages of error correction capability and supervised learning functionality. Error correction in the proposed system was applied by making a penalty function on potential regions that could be recognized as PSEPs instead of the background. Supervised learning due to the nature of deep learning made PSEP locating possible despite the different conditions of plants and the environment. This feature makes the goal of developing a generalized system for PSEPs’ detection more conceivable. Results reveal that adding the function of the penalty in the training process significantly enhanced the network effectivity, such that in all fields with different ranges, the harmonic mean of precision and recall implies the acceptable efficiency of the developed network in PSEPs’ detection.

**Figure 14:**
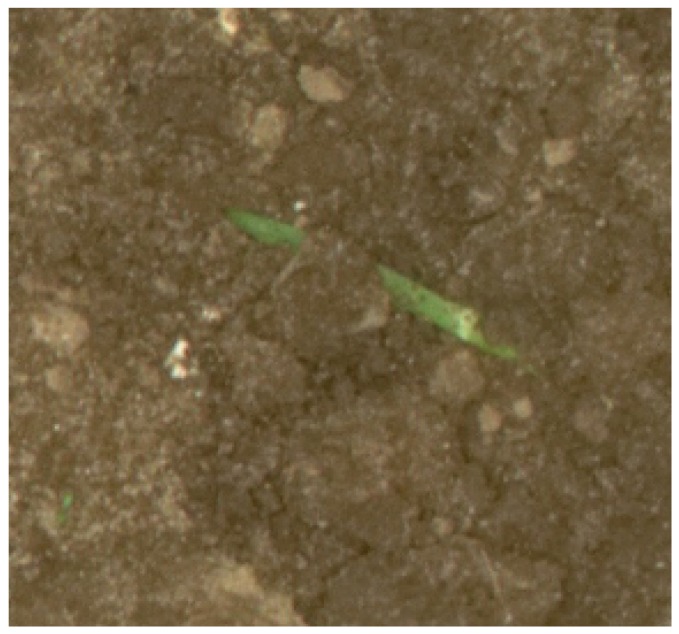
Typical image of soil clutter on the crops.

The developed PESP-locating system could be quite functional for seed drill performance evaluation. Regarding factors that would influence seeds’ placement after passing the delivery tube, evaluating the quality of sowing after the emergence of crops would provide the fairest feedback about the seed drill performance. After performing the corresponding pixel calibration, seed spacing can be determined. Based on the theoretical seed spacing and estimated ones, sowing assessment indexes included the quality of the feed index, the multiples’ index, the missed index and precision [24,25]. By discovering the exact locations of the individual crops across a field, besides evaluating the sowing performance, individual weeds identification would much be easier, such that in developing a weed recognition system, the pre-identified region of plants by the PESP-locating system can be subtracted. Then, processing field data to make a weed map without interfering crops’ effect would be achievable. Moreover, the PESP-locating system could be efficient for applying plant location-based treatments like fertilizing, thinning, etc., and controlling crop-related pests with pesticides like fungicide, nematicide, bactericide, etc. As pointed out, in some cases, identifying the exact PSEPs was hard even for the trained eye, meaning that there is some uncertainty associated with the annotations. This was because of several problems that had been found in distinguishing tips of leaves from PSEPs, the coverage of leaves from neighboring crops and soil clutter and movement on the crops (Figure 14).

**Figure 15:**
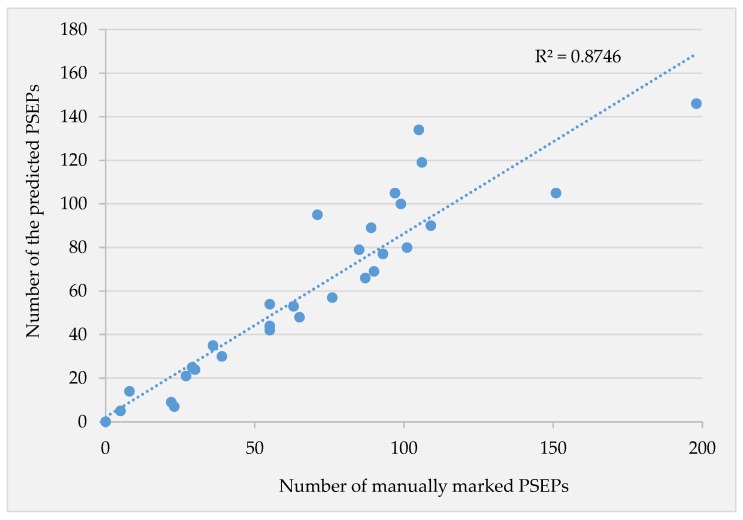
Scatter plot of the predicted number of PSEPs via counting the number of PSEPs in ground truth annotated images.

Besides all of this, the developed system showed proper functionality in counting the number of PSEPs. For applications such as sowing assessment via plant density, heterogeneous emergence in the field and plant loss due to abiotic factors, simply estimating the number of PESPs would be sufficient. The scatter plot of the predicted number of PSEPs via the number of PSEPs in ground truth annotated images is illustrated in Figure 15. The strong coefficient of determination of about 87 percent implies the appropriate ability in counting the number of PSEPs of each image. In some images, especially with denser canopies, the missed prediction and fault prediction numbers of PESPs were found to be close to each other. That caused the model to show better performance in counting the whole number of PESPs per image. The number of PSEPs in each image could refer to the number of planted seeds per unit area. This would be possible by pixel calibration during data acquisition in further studies. Along with the current study, several image processing-based studies have been previously done to identify cereal plant density [16,17]. These developed algorithms were faced with their own special constraints due to the nature of the image processing. In such machine vision-based systems, one of the most challenging obstacles is segmenting crops from the background soil properly. The application of convolutional neural networks for semantic segmentation [19,20] could be a valuable contribution for this purpose. The functionality of such an algorithm could be promoted using various conditions of soil and plants for training.

**Figure 16:**
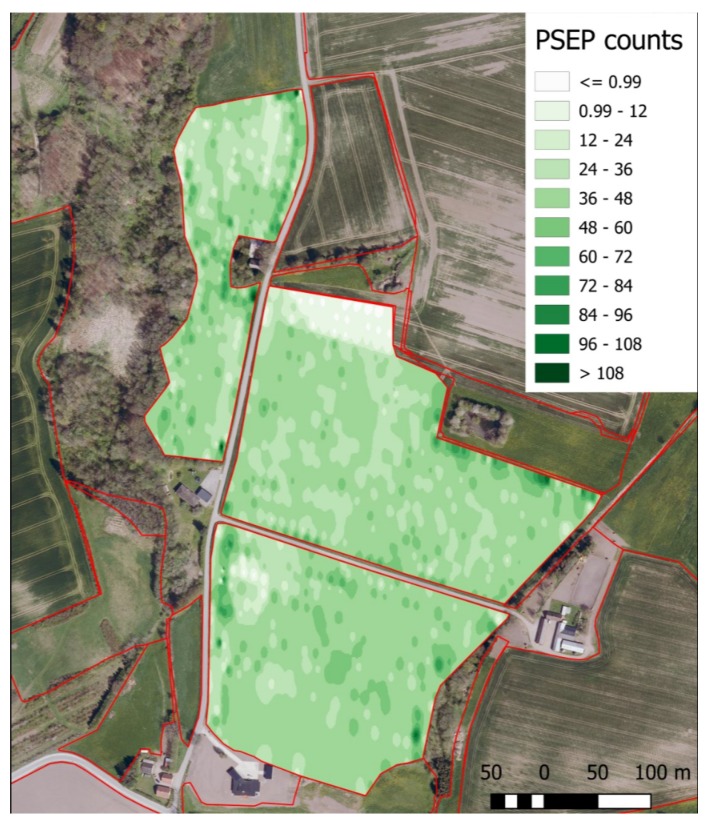
A map of the location of the predicted number of PSEPs in one of the fields in the Field 1 dataset.

During the experiments, all image data were captured by GPS position assignment. A location map of the predicted number of PSEPs in one of the fields in the Field 1 dataset is shown in Figure 16. For each captured image based on the number and range affiliation, a color has been assigned in the map. Considering the strong coefficient of determination of 87 for the evaluated images, the performance of planting in different locations can be investigated with high reliability. The map in Figure 16 could give a clue about the performance of seed drills and potential environmental factors that would have influenced the emergence rate of crops.

## 5. Conclusions

This study designed a novel and successful approach for the development of a PSEP-locating system. As several dataset from different fields were used for training, the developed model was relatively generalized in order to recognize plant location. The results revealed that the model could give acceptable feedback about sowing performance to manufacture and farmers and improve the efficiency of farm management in the further. The map of the PSEP rate alongside with a map of weeds could also give meaningful clues about the effect of planting performance on the distribution of weeds across a field. To improve such system ability in future works, the following recommendations are suggested:

- In this study, marking 212 images with possible PESPs for the training process took considerable time and required tedious work, so providing more annotated images would significantly enhance the generalized network proficiency. In future works, expanding the training dataset from various cereal fields would increase the chance of a successful application of the system in the operational phase.
- There was sometimes the problem of neighboring plants’ leaves’ coverage which made PSEPs’ identification hard, even for the trained eye. This problem was more intensive in the Field 3 dataset. To get rid of interfering leaves, data acquisition in an earlier growth stage with smaller leaves is recommended.
- In some cases, weeds were mistakenly predicted as actual crops, especially when they had a shape similar to real crops. In future studies, weeds can be subtracted from the captured image due to a pre-established map of weeds. The weeds’ map can be obtained by developing an FCN weed and crop classification model [26].
- Recently, high-resolution imagery obtained from a UAV at very low altitude was employed to develop a wheat plant density measuring system [16]. Investigating the use of UAV-based images in the developed PESP locating method in the future could be worthwhile.

## Figures and Tables

**Table 1:** A collection of 27 models compromised of three rates of the penalty (P) weights and nine softmax (SM) threshold steps.

Softmax-Thresholds								
P 00 SM 0.1	P 00 SM 0.2	P 00 SM 0.3	P 00 SM 0.4	P 00 SM 0.5	P 00 SM 0.6	P 00 SM 0.7	P 00 SM 0.8	P 00 SM 0.9
P 50 SM 0.1	P 50 SM 0.2	P 50 SM 0.3	P 50 SM 0.4	P 50 SM 0.5	P 50 SM 0.6	P 50 SM 0.7	P 50 SM 0.8	P 50 SM 0.9
P 75 SM 0.1	P 75 SM 0.2	P 75 SM 0.3	P 75 SM 0.4	P 75 SM 0.5	P 75 SM 0.6	P 75 SM 0.7	P 75 SM 0.8	P 75 SM 0.9
